# Supplementary material for: Structural variation, selection, and diversification of the NPIP gene family from the human pangenome
Source: bioRxiv. 2025 Feb 5:2025.02.04.636496. Preprint. [Version 1] doi: 10.1101/2025.02.04.636496 (PMC11838601; doi:10.1101/2025.02.04.636496)
Supplement: Supplement 2 [file NIHPP2025.02.04.636496v1-supplement-2.pdf]

bioRxiv preprint doi: https://doi.org/10.1101/2025.02.04.636496; this version posted February 5, 2025. The copyright holder for this preprint (which was not certified by peer review) is the author/funder, who has granted bioRxiv a license to display the preprint in perpetuity. It is made available under aCC-BY 4.0 International license.

SUPPLEMENTARY FIGURES

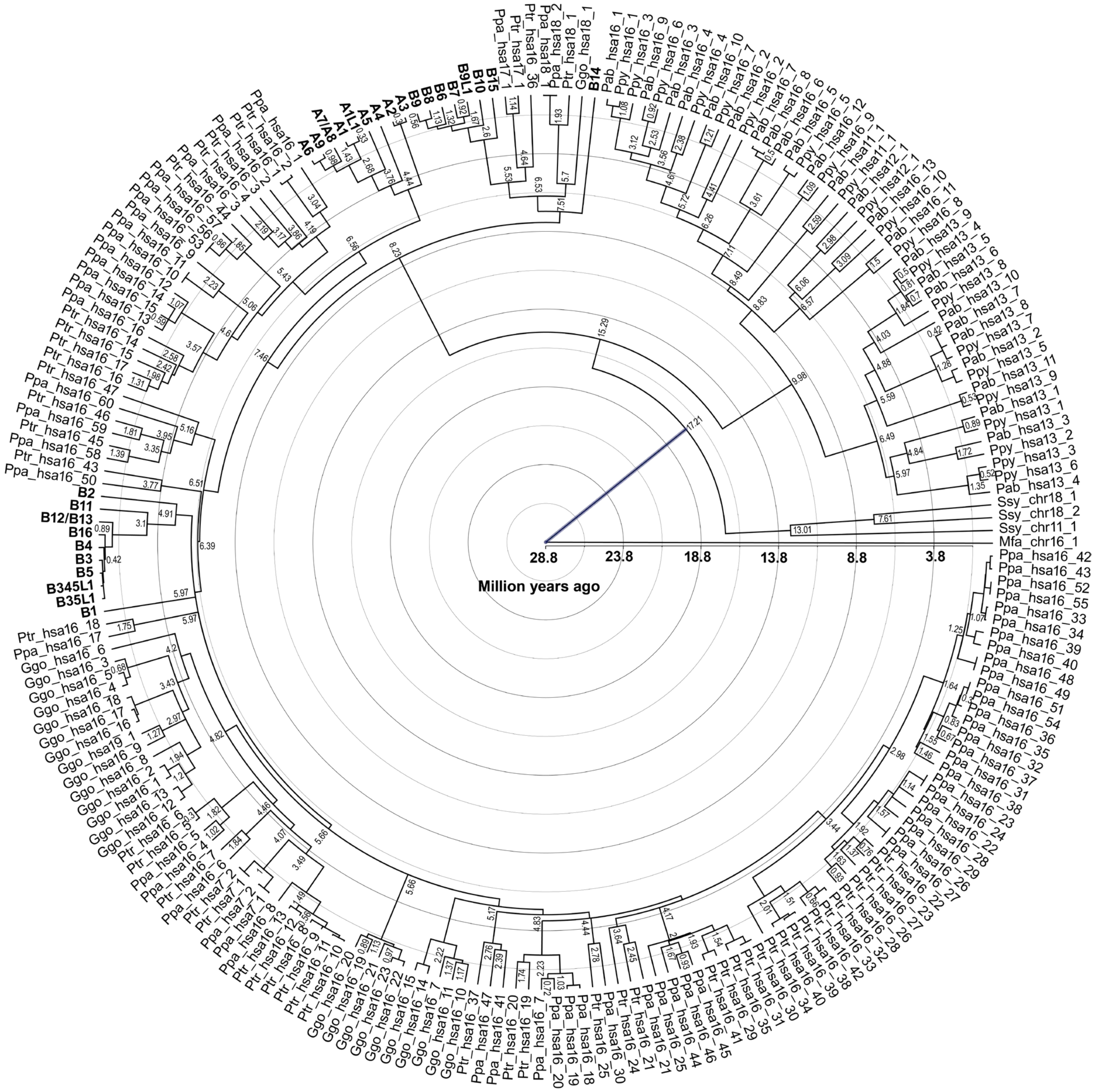

Figure S1. Timetree of human and ape *NPIP* duplications. The estimated age of *NPIP* paralogs for humans and ape species *Pan troglodytes* (Ptr), *Pan paniscus* (Ppa), *Gorilla gorilla* (Ggo), *Pongo pygmaeus* (Ppy), *Pongo abelii* (Pab), *Symphalangus syndactylus* (Ssy) is shown on a neutral phylogeny. The tree is rooted to the single copy ancestral *NPIP* from *Macaca fascicularis* (Mfa), with divergence time set to 28.8 mya. Human paralogs are bolded, and NHP paralogs are labeled by species abbreviation, chromosome number (hsa: human homologous chromosome, chr: species chromosome name), and position within the chromosome. Branch time estimates are indicated at the branch point.

bioRxiv preprint doi: https://doi.org/10.1101/2025.02.04.636496; this version posted February 5, 2025. The copyright holder for this preprint (which was not certified by peer review) is the author/funder, who has granted bioRxiv a license to display the preprint in perpetuity. It is made available under aCC-BY 4.0 International license.

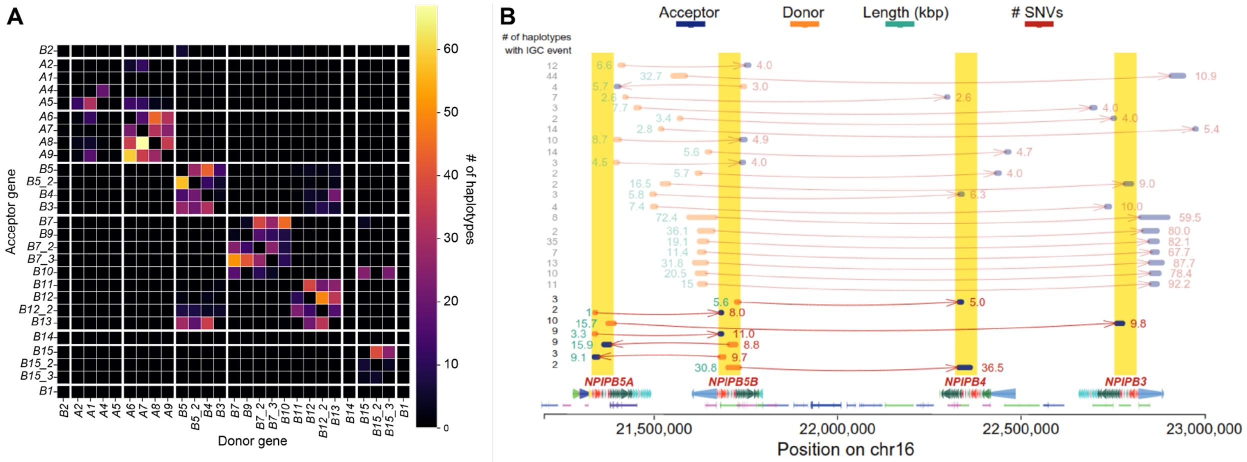

Figure S2. *NPIP* interlocus gene conversion (IGC) and structural changes. A) Counts of IGC events detected between paralogs for a subset of 94 haplotypes. X-axis labels indicate the IGC donor, and the y-axis denotes the acceptor. **B)** IGC events at the *B3-5* locus. Arrows indicate acceptors (blue) and donors (orange). The length of each event and number of SNVs are shown to the left and right, respectively.

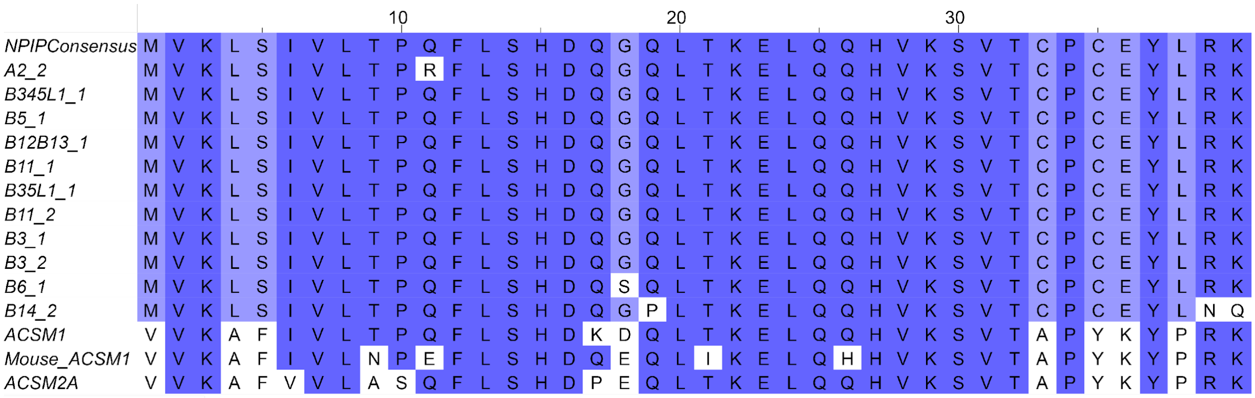

Figure S3. *NPIP* start sequence derived from *ACSM1*. Multiple sequence alignment of human *NPIP* paralog start sequences compared to human *ACSM1* and *ACSM2A* and mouse *ACSM1*.

bioRxiv preprint doi: https://doi.org/10.1101/2025.02.04.636496; this version posted February 5, 2025. The copyright holder for this preprint (which was not certified by peer review) is the author/funder, who has granted bioRxiv a license to display the preprint in perpetuity. It is made available under aCC-BY 4.0 International license.

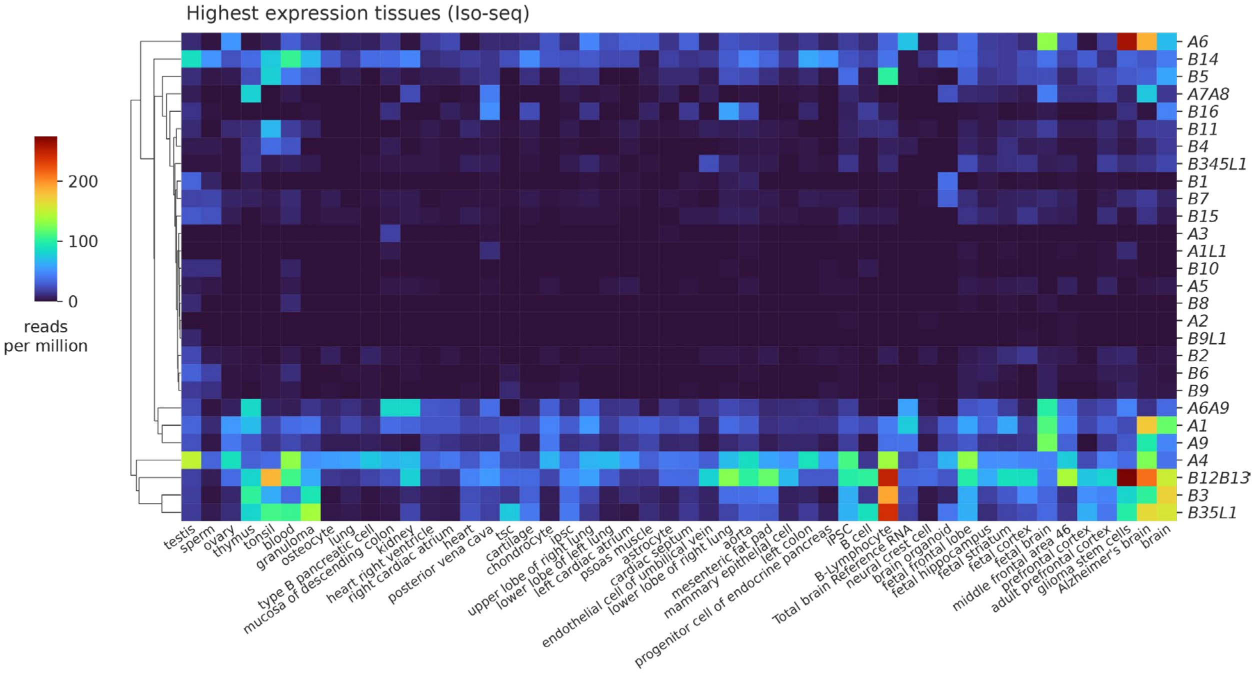

Figure S4. Variable expression of *NPIP* paralogs across tissues and cell types. Iso-Seq expression estimates for 50 tissues showing the highest *NPIP* expression, clustered with UPGMA.

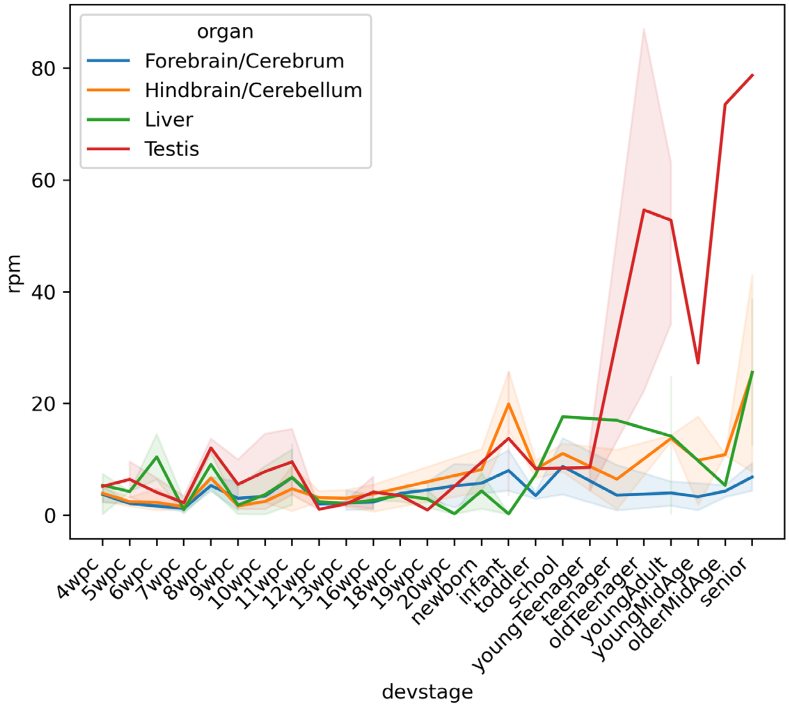

Figure S5. *NPIPB15* expression across development. Short-read expression estimates for human developmental timepoints in four tissues, using unique k-mers for paralog identity. Transparent error bands represent 95% confidence interval of replicates.

bioRxiv preprint doi: https://doi.org/10.1101/2025.02.04.636496; this version posted February 5, 2025. The copyright holder for this preprint (which was not certified by peer review) is the author/funder, who has granted bioRxiv a license to display the preprint in perpetuity. It is made available under aCC-BY 4.0 International license.

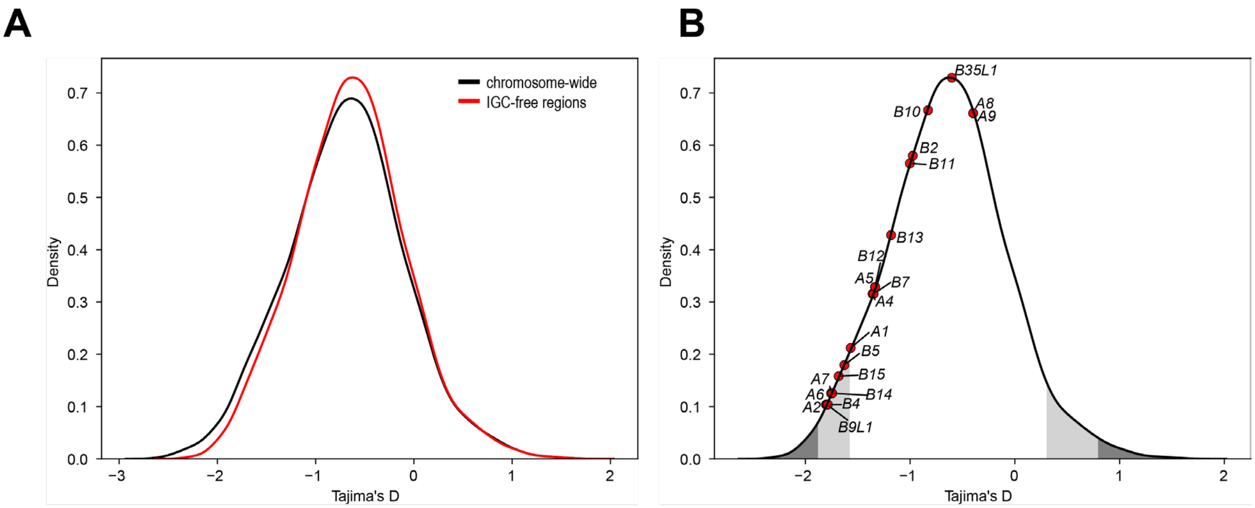

Figure S6. Tajima's D distribution in IGC-free regions. A) Tajima's D values for African individuals for the entirety of callable regions on chromosome 16 compared to IGC-free regions of chromosome 16. **B)** Tajima's D values for IGC-free windows nearest to each *NPIP* paralog. The most extreme 1% and 5%, both positive (balancing selection) and negative (positive selection) are colored in gray and dark gray.
